# Low-Threshold-For-Surgery Versus Primarily-Conservative Treatment for Odontoid Fractures in the Elderly: Evaluating Practice Variation in The Netherlands

**DOI:** 10.1177/21925682231194818

**Published:** 2023-08-08

**Authors:** Jeroen G. J. Huybregts, Samuel B. Polak, Wilco C. H. Jacobs, Ilse A. Krekels-Huijbregts, Anouk Y. J. M. Smeets, Mark P. Arts, Willem-Bart M. Slooff, F. Cumhur Öner, Wilco C. Peul, Henk van Santbrink, Carmen L. A. Vleggeert-Lankamp

**Affiliations:** 1 Department of Neurosurgery, 4501 Leiden University Medical Center, University Neurosurgical Center Holland, Leiden, The Netherlands; 2 Department of Neurosurgery, 2901 Haaglanden Medical Center, University Neurosurgical Center Holland, The Hague, The Netherlands; 3 The Health Scientist, The Hague, The Netherlands; 4 Department of Neurosurgery, 199236 Maastricht University Medical Center, Academic Neurosurgical Center Limburg, Maastricht, The Netherlands; 5 Department of Neurosurgery, Zuyderland Medical Center, Academic Neurosurgical Center Limburg, Heerlen, The Netherlands; 6 Department of Neurosurgery, 8124 University Medical Center Utrecht, Utrecht, The Netherlands; 7 Department of Orthopaedics, 8124 University Medical Center Utrecht, Utrecht, The Netherlands; 8 CAPHRI School for Public Health and Primary Care, 199236 Maastricht University, Maastricht, The Netherlands; 9 Department of Neurosurgery, Spaarne Gasthuis, Haarlem, The Netherlands

**Keywords:** odontoid process, fractures, bone, aged, cohort studies, surgical treatment, conservative treatment

## Abstract

**Study Design:**

Retrospective cohort study.

**Objectives:**

Odontoid fractures are the most common cervical spine fractures in the elderly. The optimal treatment remains controversial. The aim of this study was to compare results of a low-threshold-for-surgery strategy (surgery for dislocated fractures in relatively healthy patients) to a primarily-conservative strategy (for all patients).

**Methods:**

Patient records from 5 medical centers were reviewed for patients who met the selection criteria (e.g. age ≥55 years, type II/III odontoid fractures). Demographics, fracture types/characteristics, fracture union/stability, clinical outcome and mortality were compared. The influence of age on outcome was studied (≥55-80 vs ≥80 years).

**Results:**

A total of 173 patients were included: 120 treated with low-threshold-for-surgery (of which 22 primarily operated, and 23 secondarily) vs 53 treated primarily-conservative. No differences in demographics and fracture characteristics between the groups were identified. Fracture union (53% vs 43%) and fracture stability (90% vs 85%) at last follow-up did not differ between groups. The majority of patients (56%) achieved clinical improvement compared to baseline. Analysis of differences in clinical outcome between groups was infeasible due to data limitations. In both strategies, patients ≥80 years achieved worse union (64% vs 30%), worse stability (97% vs 77%), and – as to be expected – increased mortality <104 weeks (2% vs 22%).

**Conclusions:**

Union and stability rates did not differ between the treatment strategies. Advanced age (≥80 years) negatively influenced both radiological outcome and mortality. No cases of secondary neurological deficits were identified, suggesting that concerns for the consequences of under-treatment may be unjustified.

## Introduction

Odontoid fractures are the most common cervical spine fractures in the elderly, and their incidence is expected to further increase due to aging of the population.^1-3^ Treatment for odontoid fractures is either surgical or conservative in nature. Surgical treatment involves anterior odontoid screw fixation or (extended) posterior atlanto-axial fusion. Conservative treatment involves immobilization devices, e.g. cervical collar or halo vest. Particularly in the elderly, controversy exists about the optimal treatment as well as about the goal of treatment.

Surgical treatment carries increased risks related to the intervention and general anesthesia. Conservative treatment involves risks of prolonged fracture instability, prolonged treatment duration and complications related to immobilization. Finding a balance between fracture healing and the risk of treatment complications is challenging.^4-6^ Recent literature reviews on this topic were inconclusive, due to limited quantity and quality of the available data.^7-9^ Debate also remains as to what the treatment goal should be, because there is no convincing evidence that fracture healing clearly contributes to a more favorable clinical outcome.^10-12^ Furthermore, recent clinical studies focused on type II fractures only, while the distinction between type II and III fractures can be challenging.^13^

In the absence of high-quality evidence, the applied treatment strategies often differ between centers. The goal of this multicenter, retrospective study was to utilize this practice variation to compare the results of 2 treatment strategies: A low-threshold-for-surgery strategy (surgery for displaced fractures in relatively healthy patients, low-threshold for secondary surgery in case of prolonged instability) was compared to a primarily-conservative strategy (conservative treatment irrespective of patient/fracture characteristics). The radiological and clinical outcomes of these strategies were compared, rather than the specific treatment modalities. This approach was assumed to limit heterogeneity between groups, as no subgroups had to be selected based on applied treatment modalities. Particular focus was on the impact of age on treatment outcome (55-80 vs ≥80 years) and on cases with secondary neurological deficits. Potential prognostic factors were evaluated. Finally, the interobserver variability of the Anderson and d’Alonzo classification was studied to test the reliability of the caretakers' distinction between type II and III fractures, and to evaluate whether differences in treatment modalities derived from this distinction are – in general – appropriate.

## Methods

### Participating Centers

The authors selected 2 regions in the Netherlands with similar populations but different treatment strategies for odontoid fractures. These regions used these different strategies consistently throughout the study period.

A low-threshold-for-surgery strategy was followed in the Leiden University Medical Center (LUMC), Haaglanden Medical Center (HMC), Maastricht University Medical Center (MUMC) and Zuyderland Medical Center (ZMC). Surgical treatment was applied for dislocated fractures in relatively healthy patients, whereas conservative treatment was applied for non-dislocated fractures and patients in weak medical condition. Also, there was a low threshold for secondary surgery in case of prolonged instability or clinical symptoms (neck pain).

A primarily-conservative strategy, on the other hand, is less common and was followed in the University Medical Center Utrecht (UMCU). Conservative treatment was applied always, irrespective of fracture characteristics and the patient’s condition. Surgery was only applied as secondary treatment, in case of failure of conservative treatment.

### Patient Selection

All patients who met the selection criteria were included: 1-Patients suffered from acute (<2 weeks) type II or III odontoid fractures.^14^ 2-Patients were at least 55 years old. 3-Patients were not previously treated for odontoid fractures. 4-Patients did not suffer from systemic comorbidity expected to influence outcome (e.g. rheumatoid arthritis). 5-Surgical or conservative treatment had taken place with at least 2 weeks follow-up.

The data manager working for the LUMC and HMC conducted a sensitive search of the electronic patient files between 2000 and 2012. The data manager working for the MUMC and ZMC conducted a similar search between 2000 and 2019. The UMCU had 2 prospectively acquired databases of patients treated for spinal injuries between 2001 and 2012, from which only patients with odontoid fractures were selected. Patients from LUMC/HMC/UMCU admitted after 2012 were not included, as they were enrolled in a prospective study on odontoid fractures treatment.^15^ Patients from MUMC/ZMC were also considered for inclusion if they were admitted after 2012, as these centers were not involved in the prospective study.

The Institutional Review Boards (IRB) of MUMC and ZMC declared that the medical research involving human subjects act (WMO in Dutch) did not apply to this study (Medisch-Ethische Toetsingscommissie van het azM en Maastricht University, 2019-1280, and Medisch Ethische Toetsings Commissie van Zuyderland en Zuyd Hogeschool Zuderland, METCZ20190096, respectively). Written informed consent was obtained from all MUMC/ZMC patients. Data from LUMC/HMC/UMCU were collected in 2013, at which time IRB declarations and informed consent were not required for non-WMO studies, and data were anonymously stored since then.

### Data Collection and Analysis

Demographic parameters and fracture types from the patient files, as scored by the caretakers, were collected. Additionally, a set of review authors scored fracture types/characteristics, treatment data and outcome parameters based on a predefined data collection protocol (JH/CV for LUMC, JH/MA for HMC, JH/WS for UMCU, IH/AS/HS for MUMC/ZMC). Union was defined as evidence of bone trabeculae crossing the fracture site and absence of sclerotic borders adjacent to the fracture site on computed tomography (CT) scans. In cases of absent follow-up CT scans, union was defined as complete absence of a visible fracture line on the last follow-up X-ray. Fracture stability was defined as either presence of union or a maximum of 2 mm movement at fracture site on dynamic X-ray.^16^ Union ans stability were assessed at the last follow-up moment. Clinical outcome was retrieved from the patient files and classified as ‘clinical improvement compared to baseline’, ‘no change compared to baseline’ or ‘deterioration compared to baseline’. Fracture displacement was defined as > 2 mm displacement at the fracture site. Cases of secondary neurological deficits, secondary surgery (after failed initial treatment) and death by any cause within 104 weeks were collected.

### Statistical Analysis

Baseline characteristics and outcomes were presented using means and standard deviation (SD) for normally distributed continuous variables, and numbers and percentages for categorical variables. T-tests were done for continuous variables (age). χ^2^-tests were done for categorical variables (such as union and stability). Mann-Whitney U tests were done in case of skewed distributions (follow-up duration), of which medians and interquartile ranges (IQR) were presented. Statistical analysis of baseline American Society of Anesthesiologists (ASA) scores and clinical outcome was infeasible due to heterogeneous reporting, so they are presented descriptively. The fracture types as listed in the patient files (II/III) were used for the analysis. A Kappa (κ) value was calculated to classify the interobserver variability of the Anderson and d’Alonzo classification by comparing the original fracture score in the patient files to the independently reviewed scoring by the authors.^17^ A two-tailed *P*-value <.05 was considered statistically significant. Odds ratios (OR) and their respective 95% confidence intervals (CI) were calculated. Intention-to-treat analyses were performed using IBM SPSS, version 25.

## Results

### Patient Selection

**Figure 1.**
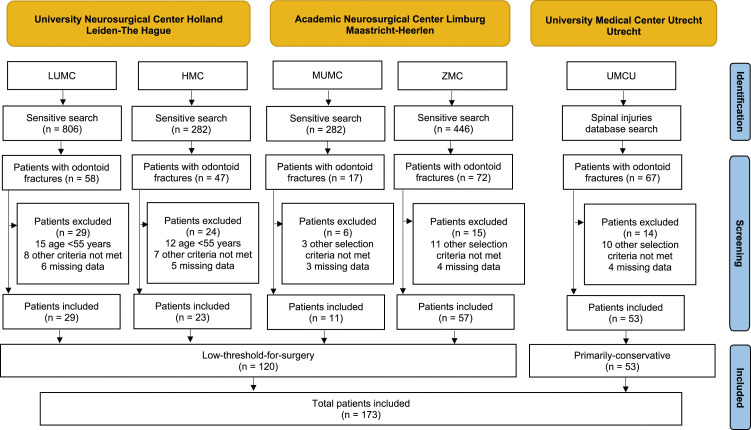
Flow diagram depicting the patient selection process.

The initial search identified 261 patients diagnosed with odontoid fractures. Of these, 88 patients did not meet the selection criteria and were excluded. The most common reasons for exclusion were age <55 years and insufficient follow-up data. A total of 173 patients were included, of whom 120 were treated with a low-threshold-for-surgery strategy and 53 with a primarily-conservative strategy (Figure 1).

### Demographic and Baseline Data

Analysis of the demographic and baseline data showed no differences between groups (Table 1). For the total cohort, mean age was 75.8 ± 10.4 years, 73 patients (42%) were ≥80 years, and 107 patients (62%) were females. Type II fractures were present in 96 patients (55%), fracture displacement was observed in 74 patients (43%), and other concomitant C1-C2 fractures were recorded in 34 patients (20%). The baseline ASA scores could be retrieved for 118 (68%) patients. Of these, 8 (7%) were ASA 1, 51 (43%) were ASA 2, 49 (42%) were ASA 3, 9 (8%) were ASA 4, and 1 (1%) was ASA 5.

**Table 1.** Summary of Demographic and Baseline Data.

	Low-threshold-for-surgery (n = 120)	Primarily-conservative (n = 53)	*P*-value
Mean age ± SD	76.6 ± 10.7	73.9 ± 9.5	.11
Age groups			
55-80 years	66 (55%)	34 (64%)	.32
≥80 years	54 (45%)	19 (36%)	
Gender			
Male	47 (39%)	19 (36%)	.74
Female	73 (61%)	34 (64%)	
Fracture type (patient files)			
Type II	69 (57%)	27 (51%)	.51
Type III	51 (43%)	26 (49%)	
Fracture displacement			
≤2 mm	72 (60%)	27 (51%)	.32
>2 mm	48 (40%)	26 (49%)	
Other C1/C2 fractures			
No	94 (78%)	45 (85%)	.41
Yes	26 (22%)	8 (15%)	

SD, standard deviation.

### Treatment Strategy

Of the 120 patients treated with the *low-threshold-for-surgery strategy*, 22 (18%) patients received primary surgical treatment: 11 (9%) underwent odontoid screw fixation and 11 (9%) underwent posterior C1-C2 fusion. The majority was primarily treated conservatively: 68 (57%) patients were treated with cervical collar and 30 (25%) with halo vest. Of the 54 patients ≥80 years (45%) in the low-threshold-for-surgery group, 8 (15%) were treated surgically.

Of the 53 patients treated with the *primarily-conservative treatment strategy*, 52 (98%) patients were treated initially conservative: 44 (83%) with halo vest and 8 (15%) with cervical collar. The remaining patient (2%) refused to undergo external immobilization and therefore primarily underwent odontoid screw fixation. None of the 19 octogenarians in the primarily-conservative group were operated.

Median follow-up duration was similar for both groups: 17 (IQR 12, 34) weeks for the low-threshold-for-surgery group and 19 (IQR 14, 37) weeks for the primarily-conservative group (Mann-Whitney *U* = 2852, *P* = .28). Secondary surgery was applied in 24 patients (20%) in the low-threshold-for-surgery group: 1 was initially treated with odontoid screw fixation, 14 initially with halo vest, and 9 initially with cervical collar. Secondary surgery was applied in 5 patients (9%) of the primarily-conservative group: 4 were initially treated with halo vest, and 1 initially with cervical collar. The mean moment for secondary surgery was 14.0 ± 12.3 weeks after start of the initial treatment.

Including cases of secondary surgery, a total of 45 (38%) patients were eventually surgically treated in the low-threshold-for-surgery group, as opposed to 6 (11%) patients in the primarily-conservative group.

### Fracture Union and Stability

No differences in fracture union and stability at last follow-up were found between the 2 groups (Table 2). Union was achieved in 63 (53%) patients in the low-threshold-for-surgery group and in 23 (43%) patients in the primarily-conservative group (OR 1.44; 95% CI .75, 2.76). Stability was achieved in 108 (90%) patients in the low-threshold-for-surgery group and in 45 (85%) patients in the primarily-conservative group (OR 1.60; 95% CI .61, 4.18). Patients aged 55-80 years achieved more union (64% vs 30%, OR 4.12; 95% CI 2.16, 7.86)) and stability (97% vs 77%, OR 9.82; 95% CI 2.76-35.0) than patients ≥80 years.

**Table 2.** Summary of the Main Results.

	Union	Stability
Overall	86/173 (50%)	153/173 (88%)
Treatment strategy		
Low-threshold-for-surgery	63/120 (53%)	108/120 (90%)
Primarily-conservative	23/53 (43%)	45/53 (85%)
OR (95% CI)	1.44 (.75, 2.76)	1.60 (.61, 4.18)
Initially applied treatment		
Surgical	15/23 (65%)	21/23 (91%)
Conservative	71/150 (47%)	132/150 (88%)
OR (95% CI)	2.38 (.92, 6.18)	1.43 (.31, 6.62)
Patient age		
55-80 years	64/100 (64%)	97/100 (97%)
≥80 years	22/73 (30%)	56/73 (77%)
OR (95% CI)	4.12 (2.16, 7.86)	9.82 (2.76, 35.0)
Gender		
Male	37/66 (56%)	60/66 (91%)
Female	49/107 (46%)	93/107 (87%)
OR (95% CI)	1.51 (.82, 2.80)	1.51 (.55, 4.13)
Fracture type		
Type II	41/96 (43%)	84/96 (88%)
Type III	45/77 (58%)	69/77 (90%)
OR (95% CI)	.53 (.29, .97)	.81 (.31, 2.10)
Fracture displacement		
≤2 mm	51/99 (52%)	86/99 (87%)
>2 mm	35/74 (47%)	67/74 (91%)
OR (95% CI)	1.18 (.65, 2.16)	.69 (.26, 1.83)
Other C1/C2 fractures		
No	72/139 (52%)	122/139 (88%)
Yes	14/34 (41%)	31/34 (91%)
OR (95% CI)	1.56 (.72, 3.28)	.69 (.19, 2.52)

OR, odds ratio; CI, confidence interval.

Union and stability were additionally evaluated separately for the 2 age groups (Table 3). For patients aged 55-80 years, union (68% vs 56%) and stability (97% vs 97%) did not differ between treatment strategy groups (OR 1.69; 95% CI .72, 3.97 and OR .97; 95% CI .09, 11.1, respectively). For patients aged ≥80 years, union (33% vs 21%) and stability (81% vs 63%) similarly did not differ between treatment strategy groups (OR 1.88; 95% CI .54, 6.48 and OR 2.57; 95% CI .81, 8.17, respectively). Median follow-up was longer for younger patients: 22 (IQR 15, 39) weeks for patients aged 55-80 years and 14 (IQR 12, 29) weeks for patients aged ≥80 years (Mann-Whitney *U* = 2661, *P* = .002).

**Table 3.** Main Radiological Outcome by Age Group.

		Union	Stability
55-80 years	Low-threshold-for-surgery	45/66 (68%)	64/66 (97%)
	Primarily-conservative	19/34 (56%)	33/34 (97%)
	OR (95% CI)	1.69 (.72, 3.97)	.97 (.09, 11.1)
≥80 years	Low-threshold-for-surgery	18/54 (33%)	44/54 (81%)
	Primarily-conservative	4/19 (21%)	12/19 (63%)
	OR (95% CI)	1.88 (.54, 6.48)	2.57 (.81, 8.17)

OR, odds ratio; CI, confidence interval.

### Clinical Outcome

Clinical outcome could be extracted from patient files for 109 patients (63%). Sixty-one (56%) patients exhibited clinical improvement compared to baseline, 25 (23%) exhibited unchanged symptoms compared to baseline, and 23 (21%) exhibited clinical deterioration compared to baseline. No cases of secondary neurological deficits were identified. Clinical outcome data were scarce in the primarily-conservative group (11%), due to the design of the database available for this group. Hence, analysis of clinical outcomes between the treatment strategies was infeasible. For the 22 surgically treated patients in the low-threshold-for-surgery group, 9 (41%) experienced clinical improvement, 2 (9%) remained the same as at baseline, and 6 (27%) experienced clinical deterioration (of which 5 aged ≥80 years), and clinical outcome could not be determined in 5 (23%) patients. Clinical outcome could be extracted in 56 (77%) patients ≥80 years. In this subgroup, 29 (52%) showed clinical improvement, 12 (21%) remained the same, and 15 (27%) showed clinical deterioration compared to baseline.

### Mortality and Complications

Death by any cause <104 weeks occurred in 12 (10%) patients in the low-threshold-for-surgery group and in 6 (11%) patients in the primarily-conservative group (OR .87; 95% CI .35, 2.46). None of these deaths could be directly related to the treatment strategy. For the 18 (10%) patients who died, mean moment of death was at 16.7 ± 14.8 weeks. Death occurred more often in the patient group aged ≥80 years: 2 (2%) patients aged 55-80 years died as opposed to 16 (22%) patients aged ≥80 years (OR .07; 95% CI .02, .33). Secondary surgery was applied in 20 (20%) patients aged 55-80 years and in 9 (12%) patients aged ≥80 years (OR 1.78; 95% CI .76, 4.17). Two patients died after secondary surgery of unrelated cause (3 and 26 weeks later, respectively). No complications were recorded in 85 (71%) patients in the low-threshold-for-surgery group and in 43 (81%) patients in the primarily-conservative group (OR .57; 95% CI .26, 1.25). No complications were recorded in 78 (78%) patients aged 55-80 years and in 50 (68%) patients aged ≥80 years (OR 1.63; 95% CI .82, 3.23).

### Prognostic Factors

#### Baseline Functioning

The baseline ASA scores could be extracted from the patient files in 118 patients (68%). Like the clinical outcome data, ASA score data were scarce in the primarily-conservative group (17%), and analysis of difference in ASA scores between groups was thus infeasible. For the 22 surgically treated patients in the low-threshold-for-surgery group, 2 (9%) were ASA 1, 11 (50%) were ASA 2, 4 (18%) were ASA 3, 3 (14%) were ASA 4, and in 2 (9%) ASA scores were missing.

#### Fracture Type

For the total cohort, less patients with type II fractures achieved union compared to patients with type III fractures (43% vs 58%, OR .53; 95% CI .29, .97). No difference was found between type II and III fractures in terms of the achievement of stability (88% vs 90%, OR .81; 95% CI .31, 2.10).

#### Fracture Displacement

For the total cohort, no influence of the presence of fracture displacement (>2 mm) was demonstrated on the achievement of union (OR 1.18; 95% CI .65, 2.16) and stability (OR .69; 95% CI .26, 1.83).

#### Other C1-C2 Fractures

For the total cohort, no influence of the presence of other C1/C2 fractures was found on the achievement of union (OR 1.54; 95% CI .72, 3.28) and stability (OR .69; 95% CI .19-2.52).

## Interobserver Variability of Fracture Type-Scoring

Blinded to the original scoring in the patient files, the authors identified 100 type II fractures and 73 type III fractures using baseline CT scans. These findings were compared to the scorings listed in the patient files. This comparison showed discrepancies in 26 (15%) of fractures. The agreement was substantial (κ = .69), indicative of the reliability of the Anderson and d’Alonzo classification (Table 4).^17^

**Table 4.** Interobserver Variability of Anderson and d’Alonzo Classification.

Kappa (κ) = .69		Fracture type according to authors (blinded scoring)	
		Type II	Type III
Fracture type according to patient files	Type II	85	11
	Type III	15	62

## Discussion

No differences in union and stability rates at last follow-up were observed between the low-threshold-for-surgery and primarily-conservative treatment strategies. The majority of patients showed clinical improvement compared to baseline. Analysis of differences in clinical outcome between treatment strategy groups or between radiological outcome groups was infeasible due to data limitations. Interestingly, no cases of secondary neurological deficits were identified, suggesting that concerns for consequences of unstable non-unions or under-treatment may be unjustified.

Patients aged 55-80 years achieved more union and stability compared to patients ≥80 years, regardless of the applied treatment strategy. In the low-threshold-for-surgery group, 18% of the total group underwent primary surgery, as opposed to 15% of patients ≥80 years. This indicates that age alone was not the decisive factor in the choice for a particular treatment modality. In both groups, mortality rates in octogenarians were higher than in non-octogenarians, which is to be expected due to life expectancy in this population. Although the common hypothesis is that treatment outcome deteriorates with advancing age, no worse clinical outcome was demonstrated for patients ≥80 years in this study.^18^

Patients with type II fractures achieved lower union rates, but similar stability rates compared to patients with type III fractures. Even though previous studies have often focused on type II fractures, the distinction between type II and III fractures is sometimes difficult to make.^1,13^ Illustratively, the interobserver analysis of fracture scoring in this study showed discrepancies in 15% of fractures. Especially for this group of fractures that does not obviously classify as either type II or III, the authors recommend caution in labeling these fractures and consequently treating them as different entities based on a sometimes debatable fracture type.

Contrary to the common presumption, the presence of odontoid fracture displacement (>2 mm) or concomitant C1/C2 fractures did not negatively influence radiological outcome. The grade of displacement may be impactful, but finding a reliable grading system is challenging in the variety of upper cervical spine fractures. Different treatment strategies were compared in this study, in which the grade of displacement was evenly distributed between both groups. These results indicate that the presence of fracture displacement or multiple fractures may be less influential on outcome than commonly thought.

### Strengths and Limitations

This patient cohort is one of the largest available so far and thereby adds to the knowledge on the topic.^7-10^ To the author’s knowledge, this is the first study in which the results of different treatment strategies (and not actual treatment modalities) were compared. This approach was used to improve comparability between groups, as considerable heterogeneity is often introduced when outcomes of surgical and conservative treatment are compared (e.g. surgery for patients in relatively good condition, conservative treatment for frail patients). The retrospective nature of this study has its associated limitations. Data was often interpreted by non-direct observers. Potential confounding variables could not be corrected for. Despite describing a relatively large cohort, this study might have been underpowered to identify potential small differences between treatment strategies (type 2 error). It can, however, be concluded that such difference, if existent, would be small and of questionable clinical relevance. Union rates at last follow-up may be an underestimation, as CT scans were not routinely made in all centers when (dynamic) X-rays showed no instability in asymptomatic patients. In such cases, to avoid false positive findings, union was only scored to be present in case of complete absence of a visible fracture line on the last X-ray. This assessment was considered less reliable than CT assessments but superior to no analysis at all. Data limitations restricted the possibilities for analysis, especially regarding clinical outcome and baseline functioning. The relatively long inclusion period was not considered influential, as treatments have not changed considerably in the last decades. Finally, in the centers that used a low-threshold-for-surgery strategy, 18% of patients were primarily operated, and another 20% underwent secondary surgery. Despite this more aggressive approach, this may still be considered relatively conservative compared to centers that may follow a primarily-surgical strategy.

### Perspective

The strategy approach used for this study allowed for a comparison between centers without need for patient sub-selection based on treatment modalities. This multicenter study examined the possible advantage of a low-threshold-for-surgery strategy (surgery for displaced fractures in relatively healthy patients, low-threshold for secondary surgery), as opposed to a primarily-conservative strategy. No evidence was demonstrated for the superiority of either one of these strategies. Prospective studies with proper adjustment for confounding and systematical evaluation of clinical outcome are needed to identify the best treatment strategy for this challenging pathology. To minimize heterogeneity introduced by patient sub-selection based on actual treatment modalities, future multicenter studies should also consider comparisons between centers, ideally comparing centers with a primarily-surgical to a primarily-conservative treatment strategy in otherwise relatively equal cohorts.

## Conclusion

This study identified no differences in union and stability rates at last follow up between low-threshold-for-surgery and primarily-conservative treatment strategies. Advanced age (≥80 years) negatively influenced radiological outcome and mortality in both treatment groups. Type II fractures resulted in lower union but comparable stability rates compared to type III fractures, even though the distinction between these entities can be difficult. No evidence was found for worse outcomes in case of dislocated or concomitant fractures. No cases of secondary neurological deficits were identified, suggesting that concerns for the consequences of unstable non-unions or under-treatment may be unjustified.

## Appendix

### Abbreviations

ASA: American Society of Anesthesiologists
CI: Confidence interval
CT: Computed tomography
HMC: Haaglanden Medical Center
IRB: Institutional review board
IQR: Interquartile range
LUMC: Leiden University Medical Center
MUMC: Maastricht University Medical Center
NA: Not applicable
OR: Odds ratio
P: *P*-value
SD: Standard deviation
UMCU: University Medical Center Utrecht
UNCH: University Neurosurgical Center Holland
WMO: Medical research involving human subjects Act (Dutch Wet Medisch-wetenschappelijk Onderzoek met mensen)
ZMC: Zuyderland Medical Center
κ: Kappa.
